# Surface-projection-based transperineal cognitive fusion targeted biopsy of the prostate: an original technique with a good cancer detection rate

**DOI:** 10.1186/s12894-019-0535-8

**Published:** 2019-11-04

**Authors:** Lei Wang, Xiaofei Wang, Wenfeng Zhao, Zichen Zhao, Zhihu Li, Shengmin Fei, He Zhu, Xiang Ji, Bing Yang, Ningchen Li, Yanqun Na

**Affiliations:** 1 Department of urology, Peking University Shougang Hospital, Peking University Health Science Center, Beijing, 100144 China; 2 Peking University Wu Jieping Urology Center, Peking University Shougang Hospital, Peking University Health Science Center, 9# Jinyuanzhuang Road, Shijingshan district, Beijing, 100144 China; 3 Department of medical imaging, Peking University Shougang Hospital, Peking University Health Science Center, Beijing, 100144 China

**Keywords:** Clinically significant prostate cancer, Targeted biopsy, Cognitive fusion biopsy, PI-RADS version 2 score, Transperineal prostate biopsy

## Abstract

**Background:**

To report a new standardized cognitive fusion technique on transperineal targeted biopsy (TB) of prostate, and to evaluate its efficacy for cancer detection combined with systematic biopsy (SB) .

**Methods:**

We present a retrospective review of consecutive patients undergoing multiparametric magnetic resonance (mpMRI) imaging of the prostate with subsequent transperineal prostate biopsy from January 2016 to December 2018. A free-hand 12-core SB was performed for each patient. PI-RADS 3–5 lesions were further targeted for biopsy with our TB technique. Firstly, a central point of suspicious lesion (B′) was registered cognitively on a transverse section of transrectal ultrasound (TRUS). Then, biopsy gun punctured vertically through a fixed pioneer site (A) on skin of perineum, and deep into the TRUS section to get A’. Next, targeted site (B), the surface-projection of B′, would be determined on skin of perineum by A and distance from B′ to A’. Finally, puncture through B to reach B′. Pathological findings of SB and TB were analyzed.

**Results:**

A total of 126 patients underwent transperineal prostate biopsy (47 SB only, 79 SB + TB). The age of the patients was 68.7 ± 9.2 years. The median preoperative PSA value was 11.8 ng/mL. Preoperative prostate volume was 60.5 ± 50.0 mL. The numbers of patients with PI-RADS scores of 1 through 5 were 4, 43, 27, 21 and 31, respectively. The overall detection rate of cancer was 61/126 (48.4%), and it was significantly higher in the combination cohort (56/79, 70.9%) compared with the SB only cohort (5/47, 10.6%, *p*<0.001). When focused on the combination cohort, TB detected a similar overall rate of PCa (53/79, 67.1% vs 52/79, 65.8%; *p* = 0.87) compared with SB. The clinically significant PCa (csPC) detection rate was 52/79 (65.8%), while for TB and SB the csPC/PC rate was 51/53 (96.2%) and 48/52 (92.3%), respectively(*p* = 0.44). TB demonstrated a better sampling performance (positive rate for each core) compared with SB (51.0% vs 31.3%, *p* < 0.001).

**Conclusions:**

Surface-projection-based transperineal cognitive fusion targeted biopsy of the prostate has a good efficacy in detecting PCa.

## Background

Prostate cancer (PCa) is one of the most frequently diagnosed malignant tumors worldwide [1]. In China, the incidence of PCa has increased rapidly in recent decades [2, 3]. Prostate biopsy is the gold standard for PCa diagnosis [4]. Hodge et al. [5]. first introduced the systematic sextant biopsy protocol under transrectal ultrasound (TRUS) guidance in 1989, and the technique of prostate biopsy has experienced a long evolution since then. Until recently, TRUS guided transperineal and transrectal biopsies are the two most frequently used approaches to obtain prostate tissue for diagnosis of PCa [6–8].

The conventional systematic method of prostate biopsy has been shown to have limited sensitivity for detecting prostate cancer in recent years [9], while multiparametric magnetic resonance imaging (mpMRI) of the prostate has become a very useful tool to improve the accuracy of prostate cancer detection. Suspicious lesions on mpMRI can guide targeted biopsies and allow for better detection of PCa, especially clinically significant PCa [10–12]. Targeted biopsy (TB) was introduced thereafter for sampling suspicious lesions shown by mpMRI. Currently, there are three primary modalities to perform a targeted biopsy: 1) MRI guided biopsy, in which biopsy is guided by real-time MRI images. This modality offers an increased sensitivity and specificity for prostate biopsy guidance; however, the disadvantages of being time-consuming and requiring specialized equipment has resulted in it not being widely used [13]. 2) MRI-TRUS fusion technique. This novel fusion technology can provide visualization of both recorded multiplanar reconstruction images on one monitor in real time, and it also produced encouraging results as reported by many researchers. However, the technology needs dedicated hardware and algorithm-based fusion software and their expense limits its widespread use [14, 15]. 3) Cognitive fusion technique, also called visual registration biopsy. This technique does not need any additional equipment but needs the operator to possess skills in reading both MRI and TURS images. It is convenient but usually requires a long learning curve [16, 17].

The cognitive fusion technique has been reported in many studies and has produced good results. However, the technique mainly relies on the operator’s personal experience and is usually not standardized and thus not easy to learn. In the present study, we introduce a new method of cognitive fusion prostate biopsy and report our experience with using this standardized and easily learned technique.

## Methods

### Study design

This study was undertaken in a single university-affiliated hospital in Beijing, China. The ethics committee of the Peking University Shougang Hospital monitored, reviewed and approved the study protocol. All consecutive men who underwent prostate biopsy from January 2016 to December 2018 were retrospectively identified. Inclusion criteria for this study were an abnormal screening test with PSA or digital rectal examination (DRE). Exclusion criteria included those men (a) with transrectal biopsy (a traditional procedure performed by an ultrasonologist in our hospital); (b) did not have an mpMRI before biopsy; (c) with a previous history of PCa; (d) with prior negative biopsy; (e) unable to communicate effectively; (f) with symptoms of urinary tract infection, acute urinary retention, or (g) coagulation disorders.

### Imaging

All of the enrolled patients underwent mpMRI on a GE Signa HDxt 3.0 T MR scanner with a 32-channel phased array coil, which included triplanar T2-weighted, axial dynamic contrast-enhanced and diffusion-weighted imaging. Image analyses were performed and supervised by two experienced uroradiologists. Suspicious lesions were assigned a likelihood score from 1 to 5 based on the standardized prostate imaging reporting and data system (PI-RADS) version 2 criteria scoring system.

### Biopsy protocol

A transperineal systematic prostate biopsy was carried out for all of the enrolled patients under intravenous or intraspinal anesthesia. A single antibiotic was routinely given 30 min before the operation. After that, a targeted biopsy was performed for suspicious lesions with a PI-RADS score of 3 to 5. Informed consent was obtained preoperatively for every patient. Details of our systematic biopsy (SB) and TB procedures are described as follows:

#### Free hand 12-core transperineal systematic biopsy

**Fig. 1:**
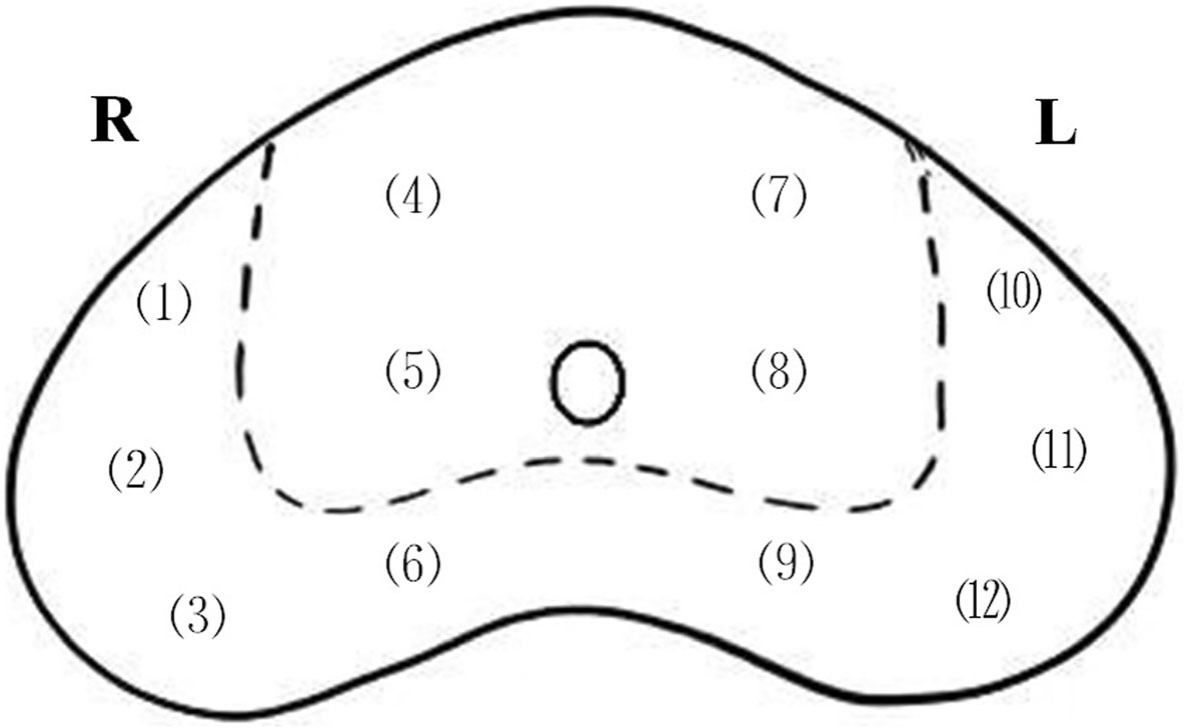
Distribution of 12 cores on a TRUS transverse section in our free-hand transperineal systematic biopsy

**Fig. 2:**
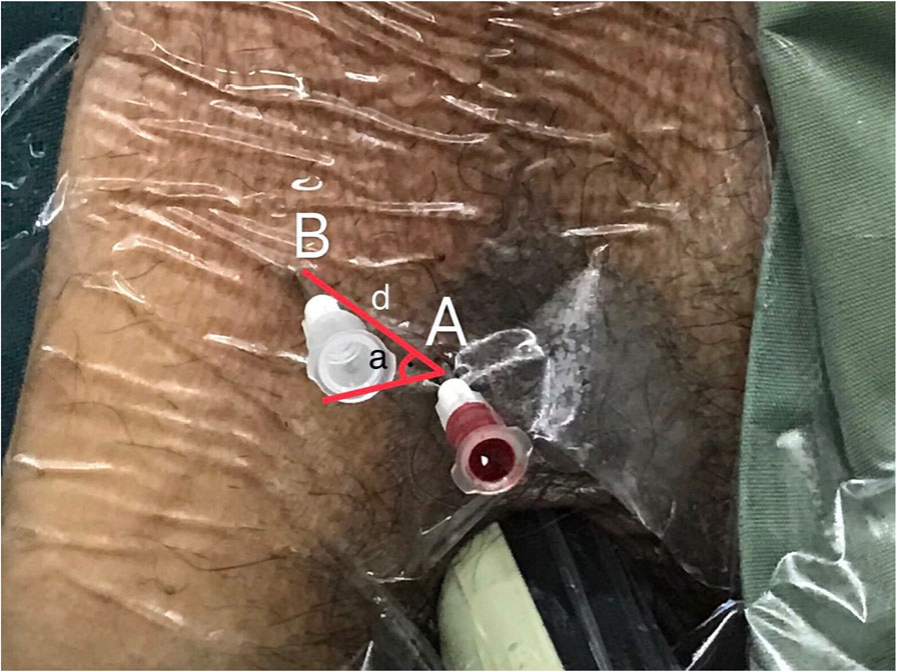
Site A and B on the perineum skin. Site A is located 1.5 cm above and 1.0 cm to the side of the middle point of the superior border of the anus. It is usually a surface projection of the middle-right region of the peripheral zone (region 6 in Fig. 1). Site B should be the surface projection of the suspicious region of the prostate. Two guiding needles have been inserted through site A and B. d: distance from B to A; a: angle from B to A

The patient was placed in the lithotomy position. A transrectal ultrasound probe (BK Medical Aps, Mileparken 34, DK-2730 Herlev) was used to obtain TRUS transversal and sagittal images simultaneously with a bipolar model. The prostate gland was divided into 12 areas on transverse sections of the TRUS image (Fig. 1). First, a puncture guided needle is located at the right side of the perineum area, approximately 1.5 cm above the superior border of the anus, 1 cm to the right of the middle line of the perineum. We call this location the pioneer site (site A in Fig. 2).

When we make a vertical puncture through site A and deep into the prostate gland, we usually reach point 6 (Fig. 1). Site A is recorded as the surface projection of point 6 on the skin of perineum. Second, an 18-G Bard disposable biopsy gun (Bard Medical, Covington, GA, USA) punctures through the guide needle, reaches the prostate gland, and then percusses to collect tissue. Third, we change the direction of the guide needle tip upward and/or to the side to obtain other tissue samples one by one. Then, the guide needle will be moved to the left side to help collect another 6 tissue samples. If the prostate gland is larger than 60 ml, another 2–8 cores will be added to provide a better coverage of the entire gland. A deeper puncture may be necessary to cover the base of the enlarged gland.

#### Surface-projection-based cognitive fusion target biopsy

**Fig. 3:**
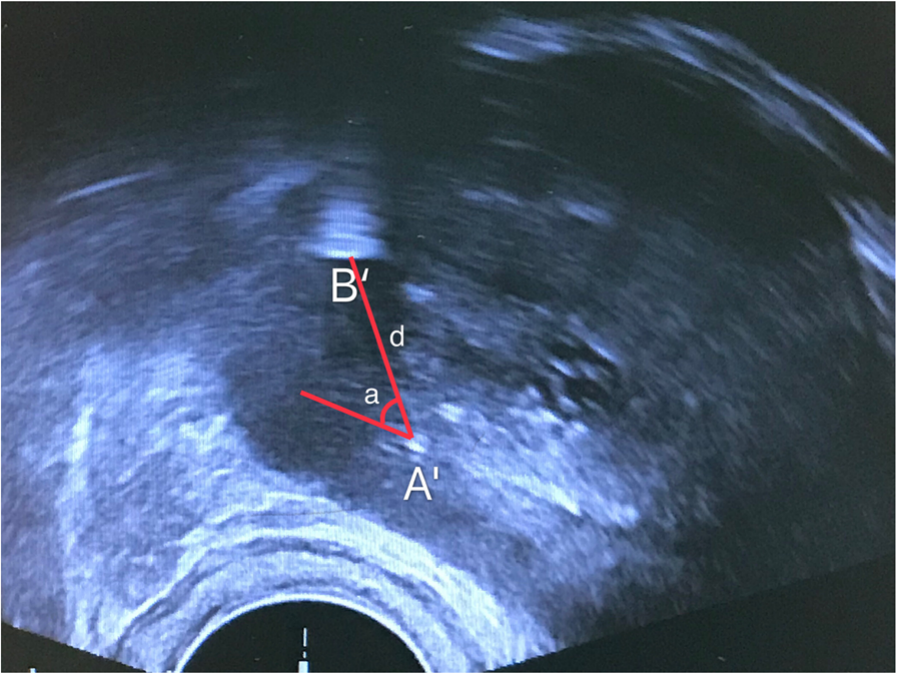
Site A’ and B′ on the target transverse section of TRUS. When the biopsy gun punctured vertically through Site A and into this target section, we can get Site A’. Site B′ represents the central point of suspicious region on the target TRUS section and it is determined by cognitive fusion of the TRUS and mpMRI images. d: distance from B′ to A’; a: angle from B′ to A’

Before our targeted biopsy, the mpMRI image is read carefully by an urologist and a radiologist together. The cancer-suspicious lesion will be identified and marked on the transverse and sagittal T2 WI sequences. The ultrasound probe is moved back-and-forth to find the same section (“*target section”*) on the TRUS transverse image as that on the MRI transverse image. After that step, four standardized steps will be performed:

Step 1: Make a vertical puncture through site A and deep into the prostate gland (the same step as that in systematic biopsy), then you will find a site representing the biopsy gun on the TRUS “target section”. We call it site A’ (Fig. 3). Site A’ in our TB protocol is actually the point 6 in our SB protocol.

Step 2: Once the location of the suspicious lesion is confirmed on the mpMRI image, it can be registered over TRUS image through fusing images cognitively. We call the suspicious lesion site B′ (Fig. 3).

Step 3: This step is to locate site B, which is the surface projection of site B′ on the skin of perineum. After the above steps, the distance (d) and angle (a) between B′ and A’ can be measured in TRUS transverse section (Fig. 3). With the same relative distance and angle from A’ to B′, we can locate site B (Fig. 2) from pioneer site A on the skin of perineum.

**Fig. 4:**
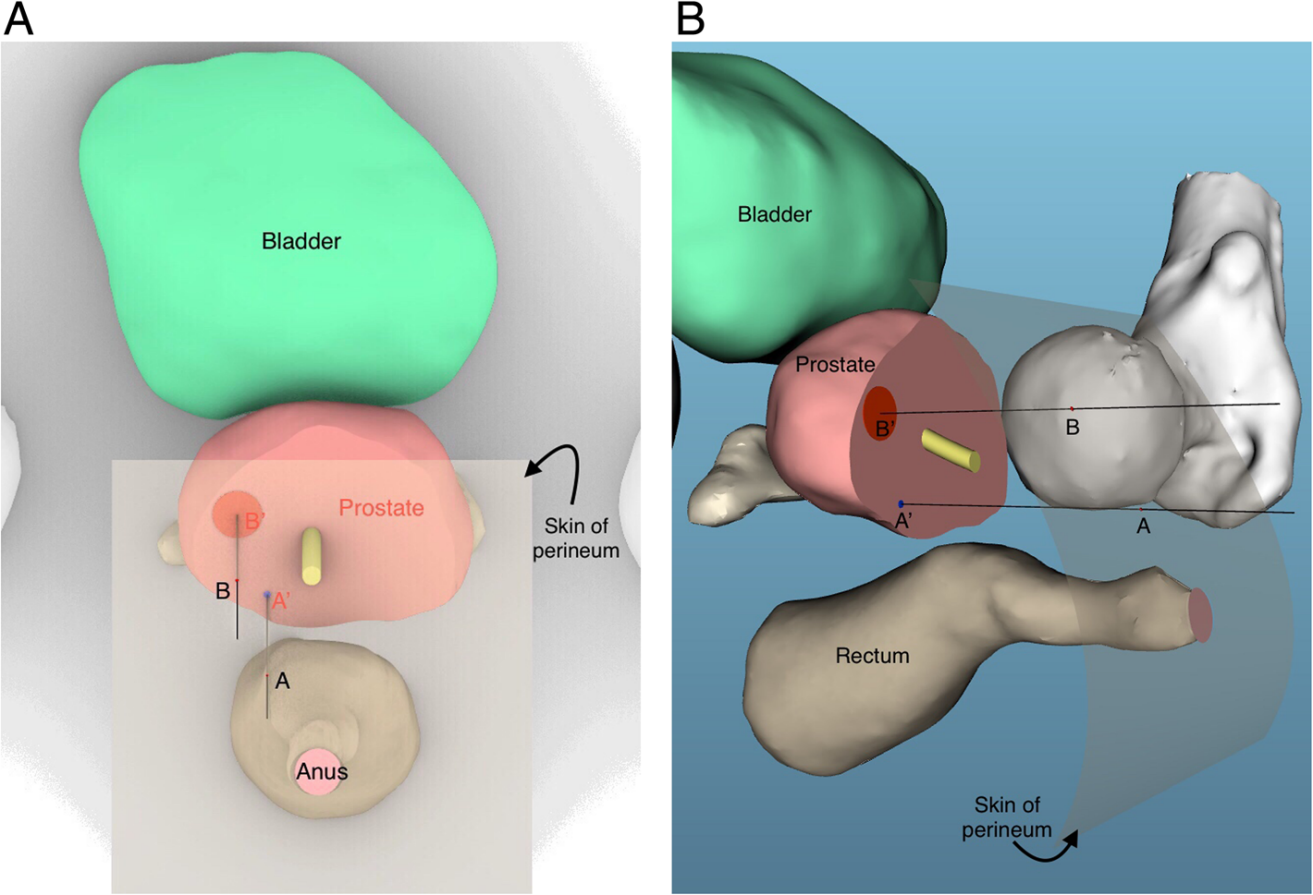
Sketch map of our technique of surface-projection-based transperineal cognitive fusion targeted biopsy of the prostate (4A: Positive View, 4B: Side View). A: a pioneer site on skin of perineum, representing the surface projection of point A’; A’: a pioneer point on transverse section of the prostate; B′: the central point of suspicious lesion; B: a targeted site on skin of perineum, representing the surface projection of point B′

Step 4: The guide needle will be inserted through site B (Fig. 2). Then, our biopsy gun will puncture through the guide needle vertically into the prostate gland to make a targeted biopsy. If the biopsy needle cannot reach the suspicious lesion precisely, the deviation should be remembered, and a slight adjustment of insert direction should be made. The number of cores sampled is mainly determined by the quality of each core, which means whether or not the core could sample the central point of the lesion, two to three samples will usually be taken for each targeted lesion. A three-dimensional image is presented as Fig. 4 to make our technique clearer.

### Clinical and pathological data

Clinical parameters involving age, DRE, PSA, prostate volume, pathological diagnosis, Gleason score, novel Gleason group, and numbers of positive cores were documented for each patient. Two pathologists independently evaluated the biopsy specimen and applied Gleason scores to the cases of PCa, and in cases of lack of consensus, a third senior pathologist evaluated the specimens. According to the novel prostate cancer grading system, we reclassified the patients by the new five grades based on the revised original Gleason score: group 1 (Gleason score ≤ 6), group 2 (Gleason score 3 + 4 = 7), group 3 (Gleason score 4 + 3 = 7), group 4 (Gleason score 8), and group 5 (Gleason score 9–10). Clinically significant cancer on biopsy was defined as a Gleason score 3 + 4 or higher or a Gleason score of 6 with positive cores ≥3.

### Data analysis

Statistical analysis was performed using SPSS 21.0 Software (SPSS Inc., Chicago, IL, USA). The quantitative data were expressed as the mean ± standard deviation when they were normally distributed or were expressed as median (inter-quartile range) when they were distributed in a skewed way. Fisher’s exact test was used to compare categorical variables. Pearson’s chi-square test was used for comparing detection rates. A *P* value< 0.05 was considered to be statistically significant.

## Results

There were 166 consecutive cases in total. Among these cases, 32 patients who underwent transrectal biopsy of the prostate, three who had no mpMRI before biopsy, and five who had prior negative biopsy were excluded. Thus, 126 cases met the criteria and were enrolled. The number of patients who underwent systematic biopsy only (SB only cohort) and who underwent systematic plus targeted biopsy (combination cohort) were 47 and 79, respectively. Among them, 42 patients had a positive DRE. The patients’ demographics and summary of biopsy findings are shown in Table 1. The mean number of targeted biopsy cores per patient was 2.5 ± 0.7. The mean number of systematic biopsy cores per patient was 12.9 ± 1.7.

**Table 1:** Patient characteristic and positive biopsy rate by different biopsy approach and PSA level

	SB only	SB + TB	*p*	All
N	47	79		126
Age	66.9 ± 8.7	69.7 ± 9.3	0.09	68.7 ± 9.2
PSA	11.5 (5.2–16.3)	13.0 (6.7–33.6)	0.07	11.8 (6.5–25.2)
Volume	72.1 ± 64.1	53.6 ± 38.2	0.045	60.5 ± 50.0
PCa	5 (10.6%)	56 (70.9%)	<0.001	61 (48.4%)
csPC	2 (4.3%)	52 (65.8%)	<0.001	54 (42.9%)
PSA (pos./tot)				
<10	2/21 (9.5%)	17/31 (54.8%)	0.001	19/52 (36.5%)
10–20	3/17 (17.6%)	11/16 (68.8%)	0.005	14/33 (42.4%)
20–50	0/8 (0%)	12/16 (75.0%)	<0.001	12/24 (50.0%)
>50	0/1 (0%)	16/16 (100%)	0.006	16/17 (94.1%)
Bone meta	1/5 (20%)	19/56 (33.9%)	0.51	20/61 (32.8%)

SB: systematic biopsy; TB: targeted biopsy; csPC:clinically significant prostate cancer; pos.:positive; tot.: total; meta: metastasis

Of the total 126 patients, 61 (48.4%) were diagnosed with PCa, including 7 (11.5%) with clinically insignificant and 54 (88.5%) with significant cancers. Fourteen (23.0%) patients had a Gleason score of 3 + 3, 12 (19.7%) of 3 + 4, 13 (21.3%) of 4 + 3, 9 (14.8%) of 8 and 13 (21.3%) patients with a Gleason score of 9–10. According to the pathological and imaging results, patients who had localized, locally advanced and metastatic prostate cancer were 25 (41.0%), 16 (26.2%) and 20 (32.8%), respectively. Complications included blood in the urine in 6 patients (4.8%), difficulty urinating in 4 patients (3.2%) and epididymitis in one patient (0.8%). Twenty PCa patients (32.8%) accepted radical prostectomy subsequently.

As shown in Table 1, patients in the SB only cohort (PI-RADS 1–2) had similar ages (66.9 yrs. vs. 69.7 yrs., *p* = 0.09), a lower PSA level (11.5 ng/ml vs. 13.0 ng/ml, *p* = 0.07), a larger prostate volume (72.1 ml vs. 53.6 ml, *p* = 0.045) and a much lower PCa detection rate (10.6% vs. 70.9%, *p*<0.001) compared with the combination cohort (PI-RADS 3–5). When stratified by PSA level, the combination cohort also manifested a much better PCa detection rate in any of the subgroups than the SB only cohort.

From Table 2, we can see that the positive rate of targeted core biopsies can reach 51.0%, which is significantly higher than that of the systematic core biopsies (31.3%, *p*<0.001). In total, 4 patients were found to have clinically insignificant prostate cancer in the combination cohort, 2 were revealed from systematic cores only, and the other 2 from both systematic and targeted cores. In combination cohort, the use of TB alone would have led to 3 fewer cases of cancer, only 1 of which was clinically significant prostate cancer (csPC). The use of SB alone would have missed 4 cases of cancer, and all were clinically significant. For TB only vs. SB only, the total and csPC detection rate were 67.1% vs. 65.8% (*p* = 0.87) and 64.6% vs. 60.8% (*p* = 0.62) and the csPC/PC rate was 96.2% vs 92.3% (*p* = 0.44).

**Table 2:** Positive biopsy rate and positive core rate in patients with systematic plus targeted biopsy

PI-RADS score	No.	PCa	PCa. only by SB	PCa. only by TB	Positive rate of Systemic Core	Positive rate of Targeted Core	*p*
3	27	8 (29.6%)	2 (1 insig.)	1	17/339 (5.0%)	7/70 (10.0%)	0.16
4	21	17 (81.0%)	1 (1 insig.)	2	63/261 (24.1%)	24/49 (49.0)	<0.001
5	31	31 (100%)	0	1	227/380 (59.7%)	68/75 (90.7%)	<0.001
Total	79	56 (70.9%)	3 (2 insig.)	4 (0 insig.)	307/980 (31.3%)	99/194 (51.0%)	<0.001

*SB* Systematic biopsy, *TB* Targeted biopsy, insig.: clinically insignificant PCa

## Discussion

TRUS guided biopsy has been proven to be an accurate method for diagnosis of prostate cancer. Additionally, with the application of mpMRI, targeted biopsy can be used to make a precise puncture. In the present study, we used an original cognitive fusion method to perform transperineal targeted prostate biopsies for patients with PI-RADS scores of 3 to 5, and this modality has been demonstrated to be easy to perform with a good PCa detection efficacy.

Targeted biopsies of the prostate under the guidance of TRUS have been widely reported with different modalities in different populations. Mischinger J and colleagues [18] reported a total and csPC detection rate of 61 and 51.9%, respectively, by using robot-assistant transperineal TB plus an SB technique in 202 patients with suspicious mpMRI. Hakozaki Y and colleagues [19] evaluated the effectiveness of MRI/US fusion TB on csPC detection and the positive rate was 57.1, 48.0, and 63.3% for SB, TB and the combination, respectively. H Lian et al. [14]. indicated that the PCa detection rates of TB, SB and combination were 30.7, 26.7 and 40.6%, respectively, by using free-hand transperineal MRI-TRUS fusion guided biopsy in Chinese men with repeated biopsy. Dekalo S et al. [20] reported their total and csPC detection rates of 52 and 35%, respectively, for 82 patients with suspicious prostate lesions on MRI by using cognitive fusion biopsy method. In our “TB + SB” cohort, the PCa detection rates of TB only, SB only and combination were 67.1, 65.8 and 70.9% respectively, while the csPC detection rate was 65.8% (Table 1). Our detection rate is higher than the results found in previous reports. The main reason for this is probably because we had more PCa patients with a higher risk and an advanced stage in this Chinese cohort. Similar to most of the previous reports, TB tended to find fewer or a similar number of overall PCas but have a higher csPC rate compared with SB in our combination cohort. For TB only, SB only and TB plus SB, the csPC/PC rates reached 96.2, 92.3 and 92.9%, respectively, in our combination cohort. However, the difference was statistically insignificant. The reason for this might be the small sample size as well as a lower rate of clinically insignificant PCa in our study. We also had a much better positive core rate for both TB and SB than in previous reports. This is mainly attributable to more advanced/high risk PCa patients in our Chinese cohort.

In the present study, as well as in other reports on Chinese patients [14, 21], there tends to be more PCa patients at high risk and in an advanced stage compared to data from western countries [15–18]. In Yang T’s biopsy cohort [21], they had 34.6% patients with a PSA level ≥ 20 ng/ml, 33.6% PCa patients with a Gleason score ≥ 8, and a total PC detection rate of 45.6%, similar to the results found in our study (32.5, 37.5, and 48.4%, respectively). We also reported 41.3% patients with PI-RADS score ≥ 4 and 32.8% PCa patients had bone metastases at first diagnosis.

Cognitive fusion targeted biopsy [16, 20, 22] has previously been reported. This kind of modality is feasible and potentially able to identify cancerous regions for subsequent biopsy through analyzing mpMRI and real-time TRUS imaging cognitively during the operation without requiring a costly real-time MRI-TRUS fusion guided biopsy system [14]. However, it is usually not easy to learn how to perform this procedure and there might be different results because of operators with different experiences. In our method, there are four important sites—site A, A’, B and B′. Site A is fixed and selected according to our previous experience in performing systematic biopsies. Site A’ can be tracked on transverse section images of TRUS as the biopsy gun punctures vertically through site A. To locate site B′, the position of the suspicious lesion, precisely on the TRUS image is the key procedure in all cognitive fusion targeted biopsies. This requires a good understanding of MRI and TRUS images and for making a mental “registration” of mpMRI images to a corresponding section of TRUS through fusing images cognitively. Finally, site B, the surface projection of site B′ on the skin of perineum, is easily selected away from the pioneer site A based on the same relative distance from A’ to B′. We believe the whole procedure is more standardized and easy to learn.

The learning curve of our technique is short and can be divided into three phases. In the first phase, the biopsy will be performed and the thought and protocol of this technique will be explained thoroughly. The trainees will visit 3–5 operations and learn how to observe the prostate gland through TURS during this period. In the second phase, the trainees will perform the biopsy by themselves under guidance. As the protocol is standardized and can be performed step by step, it is not hard to understand and operate. Most of the trainees could master this technique in 3–5 operations. After that, they will complete the biopsy independently in the last phase.

Although this method showed a good cancer detection rate in the present study, there are still several limitations. For one thing, it strictly relies on a good understanding of mpMRI and TRUS images by the operator; for another, if the suspicious lesion is smaller than 5 mm, it is hard to make a precise puncture, and more punctures should be made to increase the probability that your biopsy gun has punctured the target lesion.

For our study, there are also some shortcomings. This is a retrospective study with weak power to explain the difference of detection efficacy between the TB and SB techniques. In addition, the biopsy was performed by different operators, and there may exist an operator bias with regard to cancer detection. Finally, our study is relatively small and lacks comparisons with other methods of TB. Our data were only from one single medical center in Beijing, China, which can only partly represent the Chinese population, especially the population from northern China. A prospective and controlled study is expected to verify our new cognitive-fusion TB technique further.

## Conclusions

In the present study, we introduced a new method of cognitive fusion targeted biopsy of prostate and confirmed that it had a good performance in cancer detection. We also revealed that our targeted biopsy had better efficacy in sampling, tended to find more csPC and less clinically insignificant PCa (although a statistically difference was not reached) compared with systematic biopsy. This modality is economical and practical, and is worth to be popularized.

## Data Availability

The datasets used and analyzed during the current study are available from the corresponding author on reasonable request.

## Abbreviations

csPC: Clinically significant prostate cancer
DRE: Digital rectal examination
mpMRI: Multiparametric magnetic resonance
PCa: Prostate cancer
PI-RADS: Prostate Imaging, Reporting and Data System
PSA: Prostate specific antigen
SB: Systematic biopsy
TB: Targeted biopsy
TRUS: Transrectal ultrasound
